# Multifunctional Hard Yet Flexible Coatings Fabricated Using a Universal Step‐by‐Step Strategy

**DOI:** 10.1002/advs.202200268

**Published:** 2022-03-10

**Authors:** Yunsheng Zhang, Zixin Chen, Hao Zheng, Runze Chen, Chunfeng Ma, Guangzhao Zhang

**Affiliations:** ^1^ Faculty of Materials Science and Engineering South China University of Technology Guangzhou 510640 P. R. China

**Keywords:** anti‐biofouling, epoxy‐amine curing system, flexible hard coatings, self‐cleaning, sol‐gel chemistry

## Abstract

Hard yet flexible coatings with multi‐functionalities are useful for foldable displays and marine industries but rare. In this study, a highly cross‐linked multifunctional hybrid coating with ceramic‐like hardness and polymer‐like flexibility is reported. The coating is prepared via a step‐by‐step strategy, where two types of epoxy‐oligosiloxane nanoclusters are first synthesized by sol‐gel chemistry, and amine‐terminated curing agents are used to cross‐link them at room temperature. The coating is highly transparent (>92% transmittance), hard (6‐7H), and flexible (10 mm bending diameter) because of the unique combination of siloxane nanoclusters and polymer networks. Meanwhile, since the coating contains fouling‐resistant telomer and low‐surface‐tension liquid lubricant polydimethylsiloxane (PDMS), it exhibits excellent anti‐biofouling and self‐cleaning properties. The results indicate that the mechanical and antifouling properties of the coating can be easily tuned and prove that the step‐by‐step strategy is a promising and universal method. The novel coatings can meet the needs of applications in foldable displays, marine industries, and other fields.

## Graphical abstract

A novel multifunctional hard yet flexible coating via a universal step‐by‐step strategy has been developed. Such a coating exhibits high transparency, superior mechanical properties, and excellent anti‐fouling and self‐cleaning abilities. The coating is promising to find application in foldable displays, marine industries, and other fields.

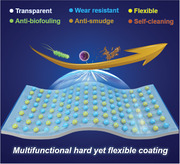

## 1 Introduction

Hard yet flexible coatings are urgent needs in foldable displays, marine industries, and other fields.^[^ ^1^ ^]^ For example, the coating for display devices or high‐speed rotating propellers needs to have a high hardness to resist wear.^[^ ^2^ ^]^ High flexibility is also required to fit curved surfaces and prevent the coating from cracking during the bending process.^[^ ^3^ ^]^ Moreover, incorporating multi‐functionality such as high transparency and self‐cleaning capabilities into flexible hard coatings is important for the rapid development of electronics display industry.^[^ ^4^ ^]^ In particular, anti‐biofouling functionality is attractive since the attachment of micro‐organisms onto device surfaces can cause serious problems,^[^ ^5^ ^]^ such as increasing surface roughness, reducing transparency, corroding device surfaces, and even spreading diseases.^[^ ^6^ ^]^

Generally, hardness and flexibility are mutually exclusive for a coating, making it difficult to achieve both properties simultaneously.^[^ ^7^ ^]^ Early coatings based on inorganic glassy materials can provide scratch and wear resistance, but they were brittle and not flexible.^[^ ^8^ ^]^ One effective solution is to develop organic–inorganic hybrid coatings by sol–gel chemistry.^[^ ^9^ ^]^ Such coatings prepared from various precursors such as functionalized silanes or metal alkoxides exhibit good mechanical properties.^[^ ^10^ ^]^ However, the thickness is limited and they require tedious production procedures. To meet the application requirements of foldable displays, coatings prepared by combining sol–gel chemistry and photocatalytic cross‐linking reaction have been developed.^[^ ^11^ ^]^ Such coatings exhibit good wear resistance and bendability but lack self‐cleaning and anti‐biofouling properties. Using a similar strategy, some omniphobic flexible hard coatings have been developed,^[^ ^12^ ^]^ which exhibit not only excellent mechanical properties but also anti‐smudge performance. Alternatively, multifunctional nanocomposite coatings have been developed by filling soft polymeric micelles into hard poly(silsesquioxane) networks, which exhibit excellent hardness, flexibility, anti‐smudge, and quick self‐healing properties.^[^ ^13^ ^]^ Note that the above coatings still lack anti‐biofouling ability and the photocatalytic cross‐linking reaction needs complex UV irradiation and limits the thickness, which in turn limits the application fields of the coatings.^[^ ^14^ ^]^ Recently, our group reported a hybrid antifouling coating consisting of an epoxy‐zirconium particle and an amine‐terminated hyperbranched polysiloxane.^[^ ^15^ ^]^ Besides superior mechanical properties, the coating simultaneously provides oil repellency and anti‐microbial abilities, which are attributed to the zwitterionic silanes. Considering that zwitterionic polymers are highly polar and hydrophilic^[^ ^16^ ^]^ and the properties of the coating can only be adjusted by redesigning the nanoclusters, it is necessary to develop a conveniently regulated strategy to meet the multi‐functionality requirements.

The epoxy‐amine curing system has been widely used in academic and industrial applications due to the simple curing conditions, high mechanical strength, and various curing agents that can easily regulate the performance of resulting coatings.^[^ ^17^ ^]^ In addition, since the epoxy‐amine reaction does not require external triggers such as UV irradiation, thick coatings can be easily prepared.^[^ ^18^ ^]^ Inspired by this, we report herein a highly cross‐linked multifunctional epoxy‐siloxane hybrid coating by combining sol–gel chemistry and epoxy‐amine curing reaction (a step‐by‐step strategy). To this end, two types of epoxy‐oligosiloxane nanoclusters (one for the matrix and the other for fouling resistance) are first synthesized by the hydrolysis and condensation reaction of epoxy‐functionalized silanes, alkoxysilanes, or silane‐terminated telomer. Subsequently, various amine‐terminated curing agents were used to cross‐link them. Last, highly cross‐linked epoxy‐siloxane hybrid coating was formed at room temperature by the reaction between epoxy groups and amino groups. The resulting coating will exhibit ceramic‐like hardness and polymer‐like flexibility because of the unique combination of siloxane nanoclusters and polymer networks. Meanwhile, since the coating contains fouling‐resistant telomer and low‐surface‐tension liquid lubricant polydimethylsiloxane (PDMS), it would exhibit excellent anti‐biofouling and self‐cleaning properties.^[^ ^19^ ^]^ We have studied the relationship between the composition of the coating and its mechanical properties, fouling resistance, and self‐cleaning abilities. We aim to develop a flexible hard coating with multi‐functionalities.

## 2 Results and Discussions

### 2.1 Universal Strategy to Prepare Hybrid Coatings

**Scheme 1:**
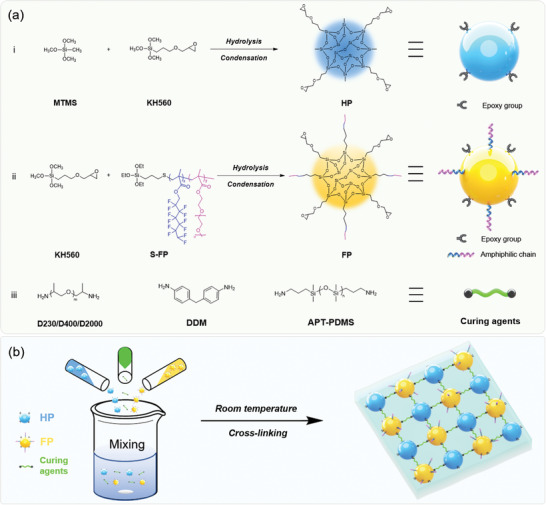
a) Preparation of hyperbranched epoxy‐oligosiloxane and fouling resistant epoxy‐oligosiloxane nanoclusters. b) Schematic description of the cross‐linking process.

**Scheme** **1** illustrates the preparation of a hyperbranched epoxy‐oligosiloxane nanocluster using 3‐glycidyloxypropyltrimethoxysilane (KH560) and methyltrimethoxysilane (MTMS), as well as the synthesis of fouling resistant epoxy‐oligosiloxane nanocluster using KH560 and silane‐terminated telomer (S‐FP). For convenience, the former is designated as HP and the latter as FP. HP and FP reacted with a variety of amino‐terminated curing agents to yield a highly cross‐linked hybrid coating at room temperature. The successful synthesis of S‐FP and epoxy‐oligosiloxane nanoclusters was confirmed via nuclear magnetic resonance spectroscopy (NMR) (Figures S1–S3, Supporting Information).

### 2.2 Influence of Different Curing Agents and Silane Precursors

To extract the roles of different curing agents on the mechanical properties of coatings, poly(propylene glycol) bis(2‐aminopropyl ether) with different chain lengths were chosen to cross‐link the HP nanoclusters. These were D230, D400, and D2000, where the numbers represent the number average molecular weights (*M*_n_). The cured coatings were designated as HP‐X, where X is the type of curing agent. Table S1, Supporting Information, shows the pencil hardness and bending diameter of the hybrid coatings. The pencil hardness of HP‐D230 or HP‐D400 is up to 7 H, indicating their surfaces have similar high hardness. However, the bending diameter of HP‐D230 and HP‐D400 is 16 mm and 12 mm respectively, indicating that HP‐D400 is more flexible than HP‐D230. As the chain length of curing agent increases, the pencil hardness of coatings decreases yet the bending diameter significantly increases. For HP‐D2000, the pencil hardness is 6 B and the bending diameter is 3 mm. Considering the application for flexible hard coatings, D400 is the optimal curing agent.

Actually, not only polyetheramines but also other types of curing agents can be used to prepare such coatings with superior wear resistance. As shown in Table S1, Supporting Information, when 4,4‐diaminodiphenylmethane (DDM) was used as curing agent, the hardness of HP‐DDM is up to 8 H, higher than that of HP‐D400, since DDM has a more rigid structure than D400. In wear resistance test, HP‐DDM was able to bear more than 1000 cycles of abrasion without visible scratches (Figure S4, Supporting Information). Other curing agents such as m‐phenylenediamine and isophorondiamine can also crosslink with epoxy‐oligosiloxane nanoclusters, the result is similar to that of DDM (Table S1, Supporting Information). In addition, using epoxy‐oligosiloxane nanoclusters synthesized by other precursors can effectively tune the mechanical properties of the coatings. A new epoxy‐oligosiloxane nanocluster designated as TPH was synthesized using tetraethylorthosilicate (TEOS), phenyltriethoxysilane (PTS), and KH560 via sol–gel chemistry. The hardness of TPH‐D400 is 8 H (Table S1, Supporting Information), higher than that of HP‐D400. The results indicate that the step‐by‐step strategy is universal and the mechanical properties of coatings can be easily tuned.

### 2.3 Preparation and Characterization of Anti‐Biofouling Coatings

To endow the hybrid coatings with anti‐biofouling abilities, we designed the amphiphilic epoxy‐oligosiloxane nanocluster (FP), where the above HP is for matrix and FP is for fouling resistant function. HP and FP were mixed in ethyl acetate and D400 was chosen to cross‐link them. The hybrid coating is designated as HPx‐FPy, where x and y represent the molar ratio of epoxy groups from HP and FP to the total epoxy groups, respectively.

**Figure 1:**
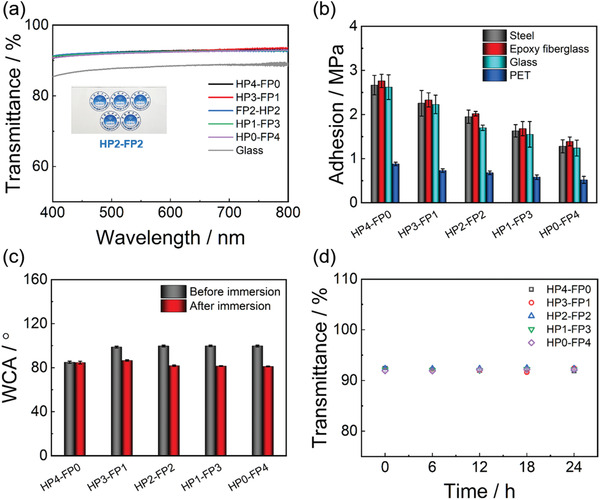
a) Transmittance spectra of HPx‐FPy coatings. b) Adhesion strength of HPx‐FPy coatings on different substrates. c) WCAs of HPx‐FPy coatings before and after immersion in water for 96 h. d) Transmittance (500 nm) of HPx‐FPy coatings after UV radiation for 24 h.

**Figure 2:**
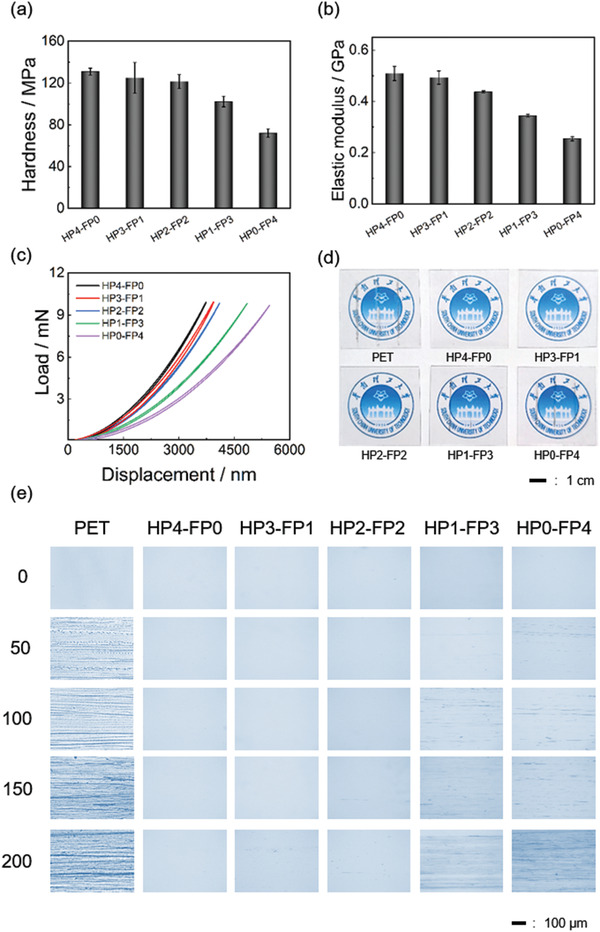
a) Hardness, b) elastic moduli, and c) load‐displacement curves of the hybrid coatings. d) Photographs of PET and the hybrid coatings after pencil scratching test. e) Optical microscope images of PET and the hybrid coatings abraded using steel wool for different cycles.

**Figure** **1**a shows transmittance spectra of the hybrid coatings coated on glass substrates. All of the hybrid coatings with a thickness of ≈60 µm exhibit similar transmittance of more than 92% from 450 to 800 nm wavelength, indicating their superior transparency. The inset in **Figure** **2**a shows the typical sample HP2‐FP2 applied to a colorful picture, the optical clarity is not reduced. Particularly, as shown in Figure S5, Supporting Information, the transmittance (500 nm) of coatings with thickness ranging from ≈60 to ≈240 µm did not vary significantly and remained above 92%, indicating the coating still maintains the high transparency as the thinness increases further. Actually, both the mixed solutions and the hybrid coatings are homogeneous, because the size of nanoclusters in the solutions and coatings is smaller than the visible light wavelength.^[^ ^20^ ^]^ Therefore, the hybrid coatings can be applied in various fields requiring high optical clarity.

Figure 1b shows adhesion strength of the hybrid coatings on various substrates. Compared to other coatings, HP4‐FP0 has the highest adhesion strength on steel (2.67 ± 0.22 MPa), epoxy fiberglass (2.76 ± 0.15 MPa), and glass (2.62 ± 0.28 MPa), indicating that the coating has strong adhesion on various substrates. This is understandable because there are hydrogen bonding and hydroxyl condensation between the hybrid coating and polar substrates.^[^ ^21^ ^]^ As the content of FP increases, the adhesion strength of coating gradually decreases such that HP0‐FP4 has the lowest adhesion on steel (1.28 ± 0.15 MPa), epoxy fiberglass (1.39 ± 0.10 MPa), and glass (1.24 ± 0.18 MPa). The reason is probably that an increase in the content of amphiphilic telomer leads to a decrease in hydrogen bond interactions. Note that all coatings have low adhesion on PET film (≈0.6 MPa), which is understandable since the surface of PET is nonpolar.^[^ ^22^ ^]^ Nonetheless, the hybrid coatings exhibit superior adhesion for applications. Actually, we can regulate the kinds of silane precuesor or curing agents to improve the adhesion of such coatings since the adhesion is mainly determined by the hydrogen bonds with the substrate.

To examine the surface wettability of the hybrid coatings and the migration of amphiphilic telomer, we used water contact angle (WCA) measurements of hybrid coatings before and after immersion in water. As shown in Figure 1c, the WCA of HP4‐FP0 is 84.9 ± 1.0° before immersion, indicating that the surface is hydrophobic. When the FP was introduced, the WCAs of hybrid coatings increased. HP3‐FP1, HP2‐FP2, HP1‐FP3, and HP0‐FP4 have WCA of 98.7 ± 0.9°, 99.7 ± 0.8°, 99.8 ± 0.7°, and 99.7 ± 0.8°, respectively, indicating that the coatings become more hydrophobic due to the introduction of FP. After immersion in water for 96 h, the WCAs of all the coatings except HP4‐FP0 decreased significantly. This is because the fluorocarbon segments with low surface energy would migrate to the surface, leading to the self‐enrichment of amphiphilic telomer on coating surface. Once contacting water, the hydrophilic PEG will in turn cover the fluorine surface. The fact is supported by X‐ray Photoelectron Spectroscopy (XPS) (Figure S6, Supporting Information). The peak at 685.7 eV is attributed to F (1s) in fluorocarbon groups. For HP4‐FP0, no F (1s) peak is detected because it does not contain any fluorocarbons. However, in the spectrum of HP3‐FP1, HP2‐FP2, HP1‐FP3, and HP0‐FP4, the F (1s) peak can be observed and the peak intensity increases gradually with the content of FP. Moreover, all the hybrid coatings except HP4‐FP0 exhibit higher F content on the surface than theoretical F content in bulk (Table S2, Supporting Information), indicating the fluorocarbon segments have a strong tendency to migrate to the surface.^[^ ^19a^ ^]^

To examine chemical stability of the coatings, we conducted tests of resistance to UV radiation and various liquids. Figure 1d shows the transmittance change of the coatings after continuous 24 h UV light irradiation. After exposure to UV radiation for 24 h, all of the coatings still have >92% average transmittance similar to their original values, indicating that they can maintain superior optical clarity. Tests of the resistance to various liquids were also conducted using a series of nonpolar organic solvents (hexane, xylene), polar organic solvents (methanol, ethanol), and aqueous solutions of acid and base (0.1 m HCl and 0.1 m NaOH, respectively). Taking HP2‐FP2 as a typical sample, none of the organic solvents or aqueous solutions influenced the pencil hardness of HP2‐FP2 and the variation in WCA is slight (Table S4, Supporting Information), indicating the coatings are greatly inert to them. The above results indicate that the coatings exhibit excellent resistance to UV radiation and various liquids.

The comprehensive mechanical performance of the coatings was examined. Figure 2a shows that HP4‐FP0 has the highest hardness, reaching 131.0 ± 3.3 MPa, which is slightly higher than that of HP3‐FP1 (124.9 ± 14.7 MPa) and HP2‐FP2 (121.5 ± 6.6 MPa). The hardness of HP1‐FP3 (102.2 ± 5.0 MPa) decreases significantly and HP0‐FP4 (72.2 ± 4.1 MPa) possesses the lowest hardness compared to other samples. This is because the introduction of amphiphilic telomer content leads to more side chains and an increase of steric hindrance.^[^ ^23^ ^]^ Thus, the coatings with more FP content have weaker mechanical properties. The elastic moduli of hybrid coatings also have a similar trend. As shown in Figure 2b, the elastic modulus of HP4‐FP0 is highest (0.509 ± 0.028 GPa) owning to its HP content being the highest. The elastic moduli of HP3‐FP1, HP2‐FP2, HP1‐FP3, and HP0‐FP4 are 0.493 ± 0.026, 0.437 ± 0.004, 0.344 ± 0.005, and 0.254 ± 0.008 GPa, respectively. As the FP content increases, the modulus of the coating decreases correspondingly. Figure 2c shows the load‐indentation curves determined by a nanoindentor. A diamond tip was pressed into the hybrid coatings at an equal loading force (10 mN). The harder the coating is, the less prone it is to be indented at the same load.

Figure 2d shows the hybrid coatings after repetitive scratching with a 6 H pencil, where a bare PET substrate was used as the control. Obviously, the surface of PET was easily destroyed by the 6 H pencil. On the contrary, no visual damages were detected on the surfaces of HP4‐FP0, HP3‐FP1, and HP2‐FP2, indicating their excellent scratch‐resistant ability. However, when the content of FP continues to increase, the surfaces of HP1‐FP3 and HP0‐FP4 were also destroyed by the 6 H pencil. This fact is further supported by the pencil hardness of coatings. As shown in Table S3, Supporting Information, HP4‐FP0 possesses the highest pencil hardness (7 H), and the introduction of FP decreases the hardness. The pencil hardness of coatings from HP3‐FP1 to HP0‐FP4 is 7 H, 6 H, 3 H, and 2 H, respectively.

The wear resistance of hybrid coatings was also examined by an industrial steel wool abrasion test using a bare PET substrate as the control, where the average pressure during test was ≈7.5 kPa. As shown in Figure 2e, numerous scratches were observed on the surface of PET after 50 cycles abrasion, indicating its poor wear resistance. For HP4‐FP0, no scratches were visible even after 200 cycles abrasion, indicating excellent wear resistance. As FP content increases, the wear resistance decreases. HP3‐FP1 can withstand 150 cycles abrasion without any scratches. In the case of HP2‐FP2, subtle scratches were observed on the coating surface after 150 cycles abrasion. For HP1‐FP3 and HP0‐FP4, obvious scratches were observed after 100 cycles abrasion. The result is consistent with the hardness and scratch‐resistance of coatings. Anyhow, the hybrid coatings still exhibit superior wear resistance compared with PET.

**Figure 3:**
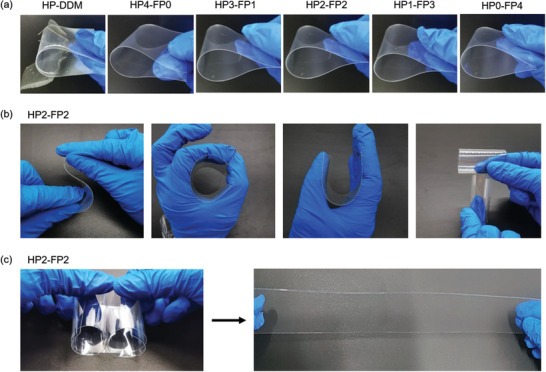
a) Photographs of the hybrid coatings after bending test. b) Photographs of HP2‐FP2 folded into “S”, “C”, “U”, “T”. c) Photographs of HP2‐FP2 after rolling up test.

We also examined the flexibility of hybrid coatings. **Figure** **3**a shows the photographs of hybrid coatings after bending test, where all of the coatings were coated on PET films with a thickness of ≈30 µm and HP‐DDM was used as a control. Significant cracks were observed on the surface of HP‐DDM after bending for 5 cycles, indicating its brittleness. In contrast, no cracks appeared on the samples of HP4‐FP0 to HP0‐FP4 even after bending for 20 cycles, indicating that the introduction of D400 can greatly improve the flexibility of hybrid coatings. We used HP2‐FP2 with high hardness as a typical sample to further examine the flexibility. As shown in Figure 3b and Figure 3c, five pieces of PET coated with HP2‐FP2 were folded into letters “S”, “C”, “U”, and “T” sequentially. No cracks appeared on the coatings during the folding process and after recovery. Then, a piece of 30 × 5 cm PET film coated with HP2‐FP2 was rolled up from both sides, and finally formed two reels with a diameter of 10 mm. The coating still did not show any cracks during the rolling process and after recovery. The flexibility of coatings was further examined quantitatively by flexibility tests (Table S3, Supporting Information). The bending diameter of HP4‐FP0 is 12 mm. As the FP content increases, the bending diameters of HP3‐FP1, HP2‐FP2, HP1‐FP3, and HP0‐FP4 are 12, 10, 10, and 8 mm, respectively, indicating that the flexibility of coatings increases correspondingly. In summary, the HPx‐FPy coatings exhibit outstanding flexibility besides their remarkable wear resistance.

**Figure 4:**
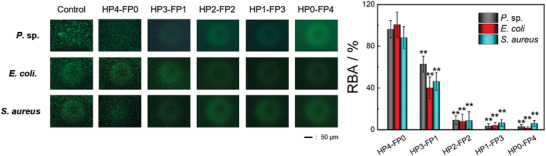
Fluorescence microscopy images of *P*. sp, *E. coli*, and *S. aureus* on the surface of hybrid coatings and relative bacteria adhesion (RBA) of the three kinds of bacteria. **p < 0.01, compared to the result of HP4‐FP0.

Antifouling ability is an important function for protective coatings.^[^ ^24^ ^]^ To investigate the synthesis process and chemical components of FP contribute to the anti‐biofouling ability of hybrid coatings, gram‐positive bacteria (*S. aureus*), gram‐negative bacteria (*E. coli*.), and marine bacteria (*Pseudomonas* sp.) were chosen as models to conduct fouling resistant assays, and a silicon wafer was used as the control. As shown in **Figure** **4**, a large number of live bacteria adhered on the surface of the control and a similar situation was observed on the surface of HP4‐FP0, indicating that they are subject to bacterial adhesion. As the FP content increased, the number of bacteria on HP3‐FP1 significantly decreased, and almost no bacteria (<10% bacterial adhesion) were observed on the surfaces of HP2‐FP2, HP1‐FP3, and HP0‐FP4. This is because the low surface energy fluorocarbon segments in FP would migrate to the surface, making the PEG self‐enriching on coating surface, which can form a hydration layer and serve as a barrier to effectively resist the biofouling.^[^ ^25^ ^]^ Clearly, the introduction of FP nanoclusters allows the hybrid coatings to have anti‐biofouling ability, which gradually improved with the increase of FP content.

### 2.4 The Introduction of APT‐PDMS for Self‐Cleaning Ability

In addition, via the step‐by‐step strategy, we can easily endow self‐cleaning ability to HPx‐FPy coatings by adjusting the curing agents. In the following experiment, HP2‐FP2 was used as the basis for its superior mechanical properties and excellent anti‐biofouling ability. Bis(3‐aminopropyl) terminated polydimethylsiloxane (APT‐PDMS) was used to replace part of the curing agents (D400) to react with epoxy‐oligosiloxanes to yield cross‐linked hybrid coatings since APT‐PDMS has low surface tension and facilitates the liquids sliding and contraction.^[^ ^26^ ^]^ Benefiting from the self‐enrichment of PDMS on the coating surface,^[^ ^27^ ^]^ only 1%, 3%, 5%, and 7% molar fraction of APT‐PDMS were used in the curing agent component. The resulting coatings are designated as HP2‐FP2‐Pz, where z is the molar percentage of APT‐PDMS in the curing agents.

Tests on the effects of the introduction of APT‐PDMS were conducted. Figure S7, Supporting Information, shows the hardness of coatings containing APT‐PDMS content. The hardness of HP2‐FP2‐P1, HP2‐FP2‐P3, HP2‐FP2‐P5, and HP2‐FP2‐P7 is 121.0 ± 7.6, 117.7 ± 7.9, 115.6 ± 2.4, and 113.7 ± 1.8 MPa, respectively. That is, the hardness decreases as the APT‐PDMS content increases. However, when the APT‐PDMS content is below 3% molar fraction, the hardness of HP2‐FP2‐P1 and HP2‐FP2‐P3 is close to that of HP2‐FP2 (122.5 ± 6.6 MPa). Meanwhile, the bending diameters of coatings do not change as the APT‐PDMS content increases (Table S5, Supporting Information). Figure S9, Supporting Information, shows the adhesion strength of hybrid coatings, revealing that the adhesion strength decreases slightly with the introduction of APT‐PDMS. In addition, the introduction of APT‐PDMS slightly reduced the anti‐biofouling ability (Figure S10, Supporting Information), but all of the coatings with APT‐PDMS still efficiently prevented most of the bacterial adhesion. The above facts indicate that the introduction of low‐content APT‐PDMS has slight effects on the mechanical and anti‐biofouling properties.

**Figure 5:**
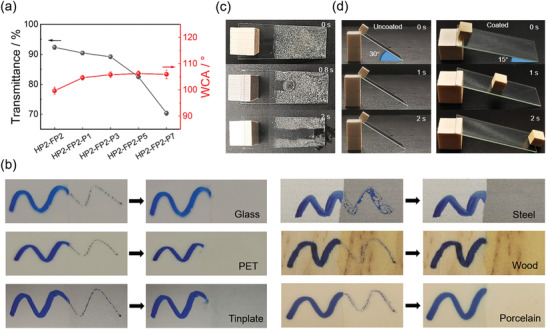
a) Transmittance and WCA variation of the hybrid coatings. b) Anti‐smudge ability of HP2‐FP2‐P3 on various substrates. c) Self‐cleaning test on a coated glass. d) Wooden cube sliding test on the uncoated and coated glass.

**Figure** **5**a shows the WCAs and transmittance (500 nm wavelength) of HP2‐FP2‐Pz coatings. Compared with HP2‐FP2 (99.7 ± 0.8°), the introduction of APT‐PDMS makes the WCAs of the coatings increase significantly. The WCA of HP2‐FP2‐P1 and HP2‐FP2‐P3 is 104.6 ± 0.9° and 105.8 ± 1.1°, respectively. However, a subsequent further increase in the PDMS content had little effect on the WCAs. The WCAs of HP2‐FP2‐P5 and HP2‐FP2‐P7 are 106.2 ± 0.9° and 105.9 ± 1.6°, respectively, similar to that of HP2‐FP2‐P3. The fact indicates that 3% molar fraction of PDMS in this system is sufficient for hydrophobicity. Furthermore, we measured the optical transmittance of the coatings with different PDMS content. As shown in Figure 5a, the transmittance of coatings decreased with increasing PDMS content. For HP2‐FP2‐P1 or HP2‐FP2‐P3, it still could retain more than 89% transmittance, but HP2‐FP2‐P5 decreased below 85%, and HP2‐FP2‐P7 decreased below 75%. That is, more than 3% APT‐PDMS content has a large impact on the transparency of the coatings. As reported before,^[^ ^28^ ^]^ PDMS has poor compatibility with basic coating and high PDMS content easily leads to phase separation, resulting in a decrease in transparency.

Figure 5b shows anti‐smudge ability of the hybrid coatings on different substrates, where HP2‐FP2‐P3 was used as a typical sample. The right half of a glass slide was coated by HP2‐FP2‐P3, while the left half was uncoated as a control. An oily pen was used to write on the glass slide. The writing on the uncoated half is clear and it is hard to be erased by a napkin. On the contrary, the writing on the coated half can be wiped off thoroughly. This is because the oil liquids fully spread out on the uncoated half but aggregated into small droplets on the coated half, and these are easy to wipe off. The similar phenomena were observed on other substrates (tinplate, PET, wood, steel, and ceramic), indicating that the coating can effectively improve the anti‐smudge ability of various substrates.

To examine self‐cleaning performance of the hybrid coatings, a glass slide coated with typical HP2‐FP2‐P3 and covered with dust was prepared. As shown in Figure 5c and Figure S12, Supporting Information, when the glass was tilted at an angle of 30°, dust can be readily removed from the coated glass via water droplets rinsing. The result of further investigation into the self‐cleaning ability of coating is shown in Figure S13, Supporting Information. Coated and uncoated tinplate were immersed in soybean oil for 12 h and then lifted. The soybean oil completely slides down the coated sample within 15 s, while a lasting oil film remains on the uncoated sample. These facts indicate that the coating has excellent self‐cleaning ability due to superior repellency toward both water and oil.

Besides anti‐smudge and self‐cleaning abilities, the hybrid coatings also exhibit surface lubricity. As shown in Figure 5d, two wooden cubes were placed on the inclined coated and uncoated glass, respectively. The wooden cube cannot slide on the uncoated glass even if the inclination angle is 30°. In contrast, the cube can easily slide off from the coated glass with an inclination angle of only 15°, indicating that the introduction of APT‐PDMS is beneficial for drag reduction. Actually, the PDMS‐containing surface also helps to prevent the attachment of fouling organisms.^[^ ^29^ ^]^ We examined the fouling release ability of coatings by pseudobarnacle tests, where an epoxy substrate and a PDMS elastomer were chosen as the control. As shown in Figure S14, Supporting Information, a remove strength as large as 1.12 ± 0.06 MPa is required on the epoxy substrate, while only 0.19 ± 0.05 MPa is required on the PDMS elastomer, indicating the excellent fouling release ability of PDMS elastomer. For HP2‐FP2, the removal strength is 0.88 ± 0.10 MPa, indicating its poor fouling release ability. As the APT‐PDMS contents increases, the values decrease. From HP2‐FP2‐P1 to HP2‐FP2‐P7, the remove strength is 0.42 ± 0.04, 0.31 ± 0.05, 0.29 ± 0.04, and 0.28 ± 0.03 MPa, respectively. Obviously, the introduction of APT‐PDMS effectively enhanced the fouling release ability. The above facts clearly indicate that the introduction of APT‐PDMS makes the hybrid coatings exhibit more functionality.

**Figure 6:**
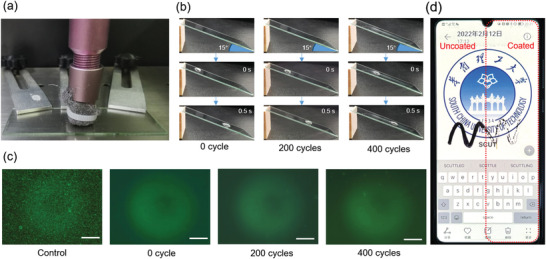
a) Photograph of the steel wool wear test. b) Water droplet sliding test of HP2‐FP2‐P3 after abrasion for different cycles. c) Fluorescence microscopy images of *S. aureus* on the surface of control and HP2‐FP2‐P3 after abrasion for different cycles. The scale bar is 50 µm. d) Photograph of HP2‐FP2‐P3 applied to a mobile phone screen.

The durability of such a coating is typically difficult and important for its application.^[^ ^30^ ^]^ To verify the durable performances of our hybrid coatings, we examined the WCA changes, self‐cleaning and anti‐biofouling properties of the coatings after different cycles of wearing test. As shown in **Figure** **6**a, a glass coated with HP2‐FP2‐P3 was subjected to wear by using steel wool for 200 and 400 cycles. After wearing the coated glass for 200 cycles, no macro scratch was observed (Figure 6b), the WCA of the coating do not change (Figure S15, Supporting Information). After wearing the coated glass for 400 cycles, some scratches were observed, and the WCA has slightly decreased, but the water droplets can still easily slide off from the tilted coating, indicating that the nonwetting or self‐cleaning performance of the coating is durable. Moreover, as shown in Figure 6c, even after abrasion for 400 cycles, the coating still exhibits excellent anti‐bacterial performance, indicating that the anti‐biofouling ability is also durable. Actually, the multifunctional coating can be simply brushed and sprayed on the surface of various devices. Figure 6d shows a case that the HP2‐FP2‐P3 coating is applied to a mobile phone screen by brushing method. Clearly, the coating exhibits high transparency and effectively prevents oil contamination, where the oil liquids are aggregated into small droplets on the coated half, and can be wiped off thoroughly (Figure S16, Supporting Information).

To meet the application requirements of foldable displays, previous organic‐inorganic hybrid coatings exhibit superior mechanical properties but usually lack anti‐smudge and anti‐biofouling abilities.^[^ ^11^ ^]^ Some omniphobic flexible hard coatings with excellent anti‐smudge ability have been developed but the anti‐biofouling problem still exists.^[^ ^12^ ^]^ To solve this problem, a zwitterion‐modified flexible hard coating has been reported,^[^ ^13^ ^]^ which has not only excellent oil repellency but also anti‐biofouling ability. Compared with zwitterion‐modified coating, the results show that the coating in this study demonstrates similarly anti‐biofouling ability but better wear resistance and anti‐smudge ability. This coating can withstand more than 150 cycles of abrasion, and the oily pen can hardly leave writings on the coating surface. In addition, compared with photocatalytic and other cross‐linking systems, the step‐by‐step strategy is more facile and universal. Although the hardness of our coating is somewhat inferior to the previous 9 H hybrid coating, the coating can be cured at room temperature without complex UV irradiation, and its mechanical and antifouling properties can be easily tuned and improved by adjusting the functionalized silanes or curing agents. Note that the step‐by‐step strategy divides traditional one‐step sol‐gel technology into the synthesis of sol and formation of gelation respectively, the reaction time, catalytic system and cross‐linked process need to be precisely controlled to obtain the target coating. Anyhow, the developed hybrid coating in this study exhibits high transparency, superior mechanical properties, excellent antifouling, and self‐cleaning abilities.

## 3 Conclusion

We have developed a multifunctional hard yet flexible hybrid coating via a simple step‐by‐step strategy. The coating exhibits high transparency, superior mechanical properties, excellent antifouling, and self‐cleaning abilities. The high hardness and flexibility arise from the combination of siloxane nanoclusters and polymer networks. The presence of amphiphilic telomer in FP allows the coating to have anti‐biofouling ability and the introduction of APT‐PDMS allows the coating to have self‐cleaning ability. Moreover, the step‐by‐step strategy is a universal method to fabricate multifunctional hybrid coating, which solves the trade‐off between high hardness and flexibility. The hybrid coatings are expected to find application in foldable displays, marine industries, and other fields.

## Conflict of Interest

The authors declare no conflict of interest.

## Supporting information

Supporting Information

## Data Availability

The data that support the findings of this study are available from the corresponding author upon reasonable request.
