# Supplementary material for: Multifunctional Hard Yet Flexible Coatings Fabricated Using a Universal Step‐by‐Step Strategy
Source: Adv Sci (Weinh). 2022 Mar 10;9(14):2200268. doi: 10.1002/advs.202200268 (PMC9109054; doi:10.1002/advs.202200268)
Supplement: Supplementary file 1 — Supporting Information [file ADVS-9-2200268-s001.pdf]

ADVANCED SCIENCE
Open Access

Supporting Information

for *Adv. Sci.*, DOI 10.1002/advs.202200268

Multifunctional Hard Yet Flexible Coatings Fabricated Using a Universal Step-by-Step Strategy

Yunsheng Zhang, Zixin Chen, Hao Zheng, Runze Chen, Chunfeng Ma and Guangzhao Zhang*

Supporting Information

Multifunctional Hard yet Flexible Coatings Fabricated Using a Universal Step-by-step Strategy

Yunsheng Zhang, Zixin Chen, Hao Zheng, Runze Chen, Chunfeng Ma, and Guangzhao Zhang*

Y. Zhang, Z. Chen, H. Zhen, R. Chen, Prof. C. Ma, Prof. G. Zhang
Faculty of Materials Science and Engineering, South China University of Technology, Guangzhou 510640, P. R. China
E-mail: msmcf@scut.edu.cn

1. Experimental Section

Materials

Dodecafluoroheptyl methacrylate (DFMA), poly(ethylene glycol) methyl ether methacrylate (PEGMA, M_n = 475 g/mol), 3-mercaptopropyl triethoxysilane (KH580), 3-glycidyloxypropyltrimethoxysilane (KH560), methyltrimethoxysilane (MTMS), tetraethyl orthosilicate (TEOS), phenyltriethoxysilane (PTS), poly(propylene glycol) bis(2-aminopropyl ether) (D230, D400, and D2000, M_n = 230 g/mol, 400 g/mol, and 2000 g/mol, respectively), ethyl acetate, 4,4-diaminodiphenyl methane (DDM), m-phenylenediamine (MPD) and isophorondiamine (IPDA) were from Aladdin and used as received. Azobisisobutyronitrile (AIBN) from Aladdin was purified by recrystallizing in methanol. Bis(3-aminopropyl) terminated polydimethylsiloxane (APT-PDMS) from Macklin was used as received. Hydrogen chloride solution (HCl, 37 wt%), ethanol (EtOH), and methanol were from Sinopharm and used as received. Tetrahydrofuran (THF) and hexane were from Sinopharm and distilled by CaH_2. Xylene from Guangzhou Chemical Reagent Factory was used as received. PDMS elastomer (Sylgard 184) was purchased from Dow Corning.

Synthesis of epoxy-oligosiloxane nanoclusters

Hyperbranched epoxy-oligosiloxane nanocluster: KH560 (23.6 g), MTMS (9.0 g), H_2O (6.1 g), HCl solution (0.3 g), and ethanol (20 mL) were mixed in three-neck flask with a magnetic stirrer. The solution was allowed to hydrolyze at 60 °C for 8 h, then it was concentrated under vacuum to remove volatiles. The obtained nanocluster was designated as HP. Using a similar protocol, another epoxy-oligosiloxane nanocluster was synthesized by using KH560 (23.6 g), TEOS (6.9 g), PTS (16.0 g), H_2O (6.8 g), HCl solution (0.4 g), and ethanol (20 mL), which was designated as TPH.

Fouling resistant epoxy-oligosiloxane nanocluster: Silane-terminated telomer (S-FP) was synthesized according to the literature.[1] KH560 (23.6 g), H_2O (3.1 g), HCl solution (0.2 g), and ethanol (20 mL) were stirred at 60 °C for 4 h. Then S-FP (9.0 g), H_2O (0.2 g), and HCl

solution (0.1 g) were added to the mixture, which was continued to be hydrolyzed for another 4 h. After being concentrated under vacuum to remove volatiles, the fouling resistant epoxy-oligosiloxane nanocluster was obtained, which was designated as FP.

Preparation of the hybrid coatings

The hybrid coatings were prepared by using different curing agents to cross-link the HP and FP nanoclusters. Typically, HP2-FP2 was prepared by mixing HP (1.3 g), FP (1.5 g) and D400 (1.0 g) in ethyl acetate (4 mL), then the mixture was stirred at 25 °C for 10 min. After stirring, the reacted solution was dropped on different substrates and dried at 25 °C for 7 d to ensure complete curing. When using APT-PDMS to replace part of the curing agents, APT-PDMS should be mixed with nanoclusters first and stirred at 25 °C for 6 h, then D400 was added to prepare the coatings. Following the similar protocol, other hybrid coatings were prepared.

2. Characterization

Nuclear Magnetic Resonance Spectroscopy (NMR). ^{1}H NMR Spectrum and ^{29}Si NMR spectra were performed on a Bruker AV600 NMR Spectrometer using $CDCl_3$ as the solvent and tetramethylsilane as the internal standard.

Pencil Hardness Tests. The pencil hardness of coating was measured according to the ASTM D3363 (2020).[2] Pencils with hardness ranging from 6 B (The softest) to 9 H (The hardest) were used to scratch along the coating surface at an angle of 45°. The pencil hardness of coating was equivalent to that of the hardest pencil that did not leave a scratch on the coating surface.

Flexibility Tests. The flexibility test was conducted according to GB/T1731-93[3] by a coating flexibility tester (Shanghai Jingge Technology Co., Ltd, Model QTX). All of the coatings were coated on tinplate and bent on shaft rods of different diameters. The flexibility was

determined by the minimum diameter of the shaft rod that did not make the coating crack after bending.

Transmittance Spectra Measurements. All samples were coated on cleaned glass substrate with a thickness of ~60 μm. The UV-Vis spectra were recorded by a UV-Vis spectrometer (Agilent, Model Cary 3500).

Adhesion Tests. The adhesion tests were performed by an automatic tester (PosiTest, Model AT-A) according to ASTM D4541-09.[4] All samples were coated on steel, epoxy fiberglass, glass, and PET film for adhesion tests. Before test, the coating surface was polished lightly by abrasive paper to make the aluminum studs adhere better. The rate of pull was set to 0.2 MPa/s. The average adhesion strength was calculated based on 6 different regions of the coatings.

Surface Wettability Measurements. The water contact angles (WCAs) of the coatings were measured by dropping 3 μl water on the surface of the coatings at 25 °C using a contact angle goniometer (Biolin, Model KSV NIMA). Each average value was calculated from 5 different regions of samples.

X-ray Photoelectron Spectroscopy (XPS). XPS analysis was performed using an Axis Ultra DLD (Kratos Analytical) with a monochromate Al Kα (hν = 1486.6 eV) source at takeoff angles of 90° to investigate the surface compositions.

UV Resistance Tests. The UV resistance of the coatings was measured by a QUV accelerated weathering tester (Q-Panel Lab, Model QUV/spray) according to ASTM G154.[5] All the coatings coated on glass were fixed in the instrument and exposed to UV irradiation for 24 h. The test intensity was 0.83 W/m^2 and the test temperature was 50 °C. The transmittance spectra, WCAs, and pencil hardness of the coatings were measured before and after exposure. Tests were performed in triplicate.

Liquids Resistance Tests. The liquid resistance of the coatings was conducted referring to ISO 2812-1-2017.[6] Hexane, xylene, methanol, ethanol, 0.1 M HCl and 0.1 M NaOH aqueous

solutions were used as the test liquids. All the coatings coated on glass plate were completely immersed in the test liquids for 24 h at 25 °C, and then placed in the oven at 60 °C for 4 h to remove the residual liquid. The WCAs and pencil hardness of the coatings were measured before and after immersion. Tests were performed in triplicate.

Nanoindentation Tests. A nanoindentor (Anton Paar, Model TTX-NHT3) equipped with Berkovich diamond tip was used for nanoindentation tests at 25 °C. Oliver-Pharr method was used to quantify the hardness (H_{IT}) and elastic modulus (E_{IT}). The loading and unloading rate were both set to 10.00 mN/min. All of the samples were coated at glass substrate with thickness of ~100 μm and their hardness (H_{IT}) and elastic modulus (E_{IT}) were calculated by computer from loading-displacement curve.[7]

Scratch Resistant Tests. A 6 H pencil was used to scratch on the surface of bare PET substrate and hybrid coatings. After 20 cycles of scratching, the scratches on the surface of all samples were recorded.

Wear Tests. Abrasion tests were performed on an Abrasion Resistance Tester (ZL-7013B). All of the samples were fixed on a horizontal board, then a test rod with a moving arm was set to compress a steel wool mat tightly on the sample. Abrasion was realized by the movement of arm and thus the steel wool mat can run back and forth on the coating surface at a rate of 20 r/min. After abrasion, the surface wear of the coatings was recorded using an optical microscope.

Bending Tests. All of the coatings were coated on PET substrate with a thickness of ~30 μm. The coated film was bent to a U shape and then released. The bending and releasing processes were repeated 20 times.

Folding and Rolling up test. HP2-FP2 was coated on 5 short PET films (10 × 5 cm) and a long PET film (30 × 5 cm). The former was folded into letters “S”, “C”, “U” and “T” sequentially, and the latter was rolled up from both sides, and finally formed two reels. Tests were performed in triplicate.

Anti-biofouling Assays. Gram-positive bacteria (*S. aureus*), Gram-negative bacteria (*E. coli.*) and marine bacteria (*Pseudomonas* sp.) were used to characterize the broad-spectrum antibacterial ability of hybrid coatings. Typically, S. *aureus* was cultured on Tryptic Soy Agar (TSA) plates at 37 °C for 24 h. Then, monoclonal colonies were inoculated into 5 mL Trypsin Soy Broth (TSB) solution and grown at 37 °C. Following a similar protocol, the marine bacteria *Pseudomonas* sp. and E. *coli* were cultured. Before tests, 1 × 1 cm silicon slides coated with samples were immersed in 1 mL of bacterial suspension (10^8 cells/mL) for 4 h at 30 °C in polystyrene 24-well tissue culture plate. Subsequently, the bacteria were dyed by 3 μl LIVE/DEAD BacLight Bacterial Viability Kit for 30 min and washed twice to wash out the planktonic bacteria. After that, a fluorescent microscope (Zeiss, Model Scope A1) was used to observe the dyed bacteria on coatings.

Anti-smudge test. The hybrid coatings were coated on the right half of different substrates (glass, tinplate, PET, wood, steel, and ceramic) while the left half of substrates was bare. An oily pen was used to write on the surface and the writings were observed. Then, napkin was used to wipe off the writings, the erasure of writings was recorded.

Self-cleaning test. Dust was covered on the surface of a coated glass. Then, water droplets were dropped on the coating surface to remove dust, the removal of dust was recorded.

Oil sliding test. Uncoated tinplate and coated tinplate was immersed in soybean oil for 12 h and lifted up, respectively. The time of all soybean oil slipped from the tinplate was recorded.

Surface lubricity test. Two wooden cubes were placed on the inclined coated and uncoated glass to slide off, respectively. The uncoated glass was inclined at 30° and the coated glass was inclined at 15°. The sliding time of cubes from glass top to bottom was recorded.

Pseudobarnacle removal tests. Following the standard of ASTM D5610 (2011),[8] 5 cylindrical aluminum studs (10 mm diameter) were glued on the coating surface using epoxy adhesive (Araldite). A shear force parallel to the surface was applied to the studs using a force gauge (SUNDOO, Model SH-500) to measure the removing strength.

Statistical analysis. Data are expressed as the mean ± standard deviation (SD). Statistical analysis was performed using one-way ANOVA testing. Significance was defined as $p < 0.05$.

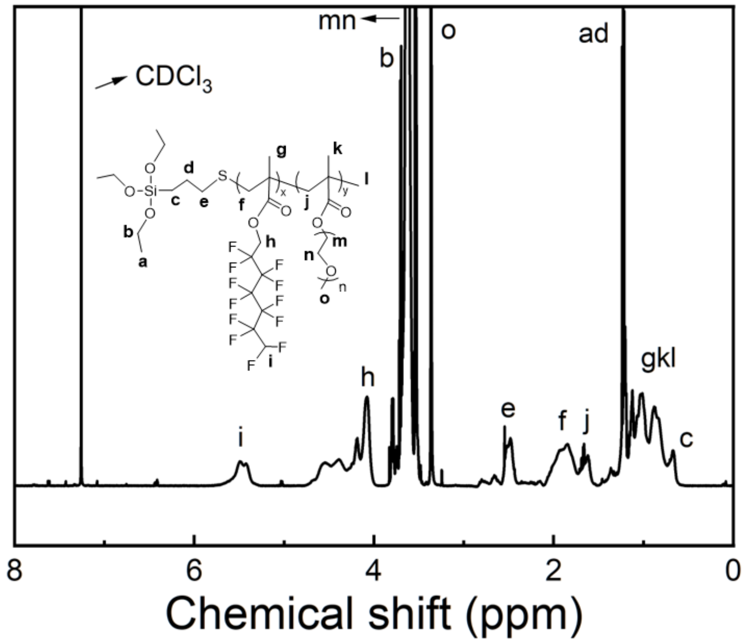

Figure S1. ^{1}H NMR spectrum of S-FP.

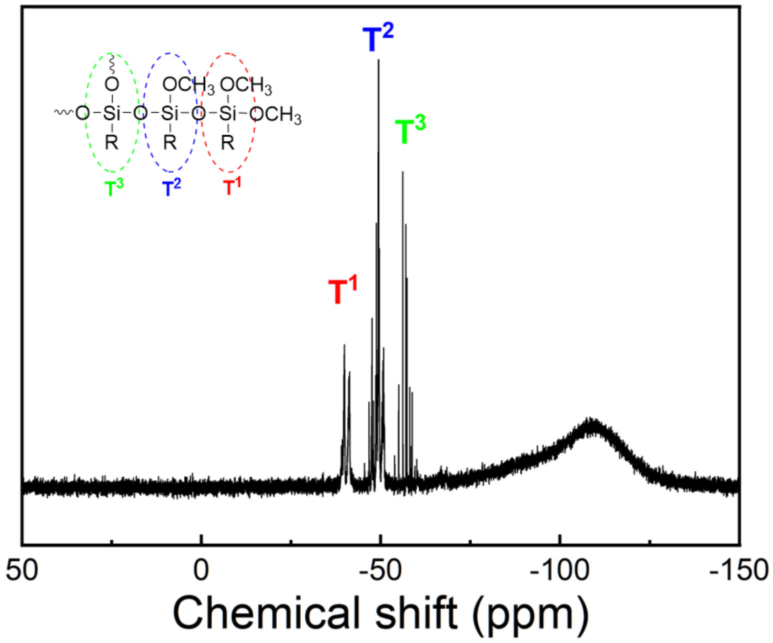

Figure S2. ^{29}Si NMR spectrum of HP.

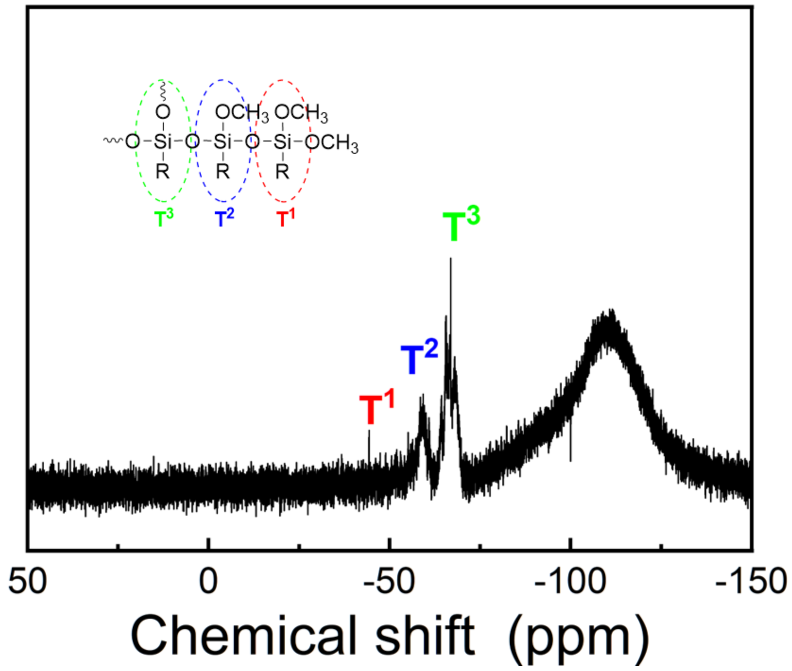

Figure S3. ^{29}Si NMR spectrum of FP.

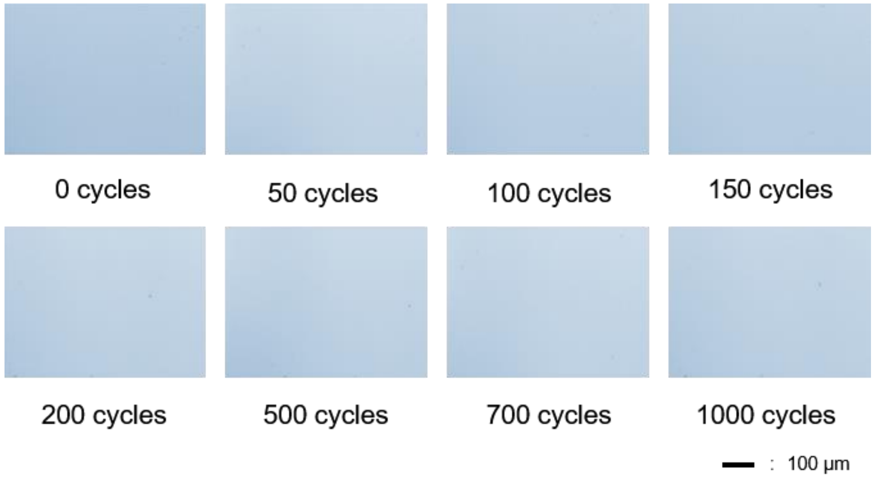

Figure S4. Optical microscope images of HP-DDM after abrasion tests.

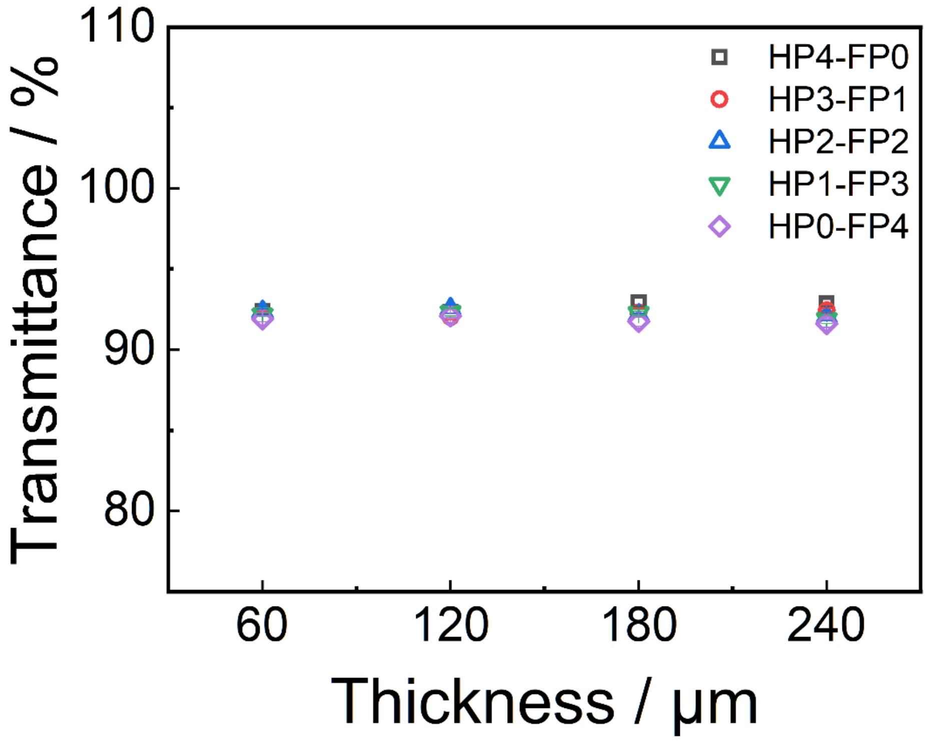

Figure S5. Thickness dependence of the average transmittance (500 nm) of the hybrid coatings.

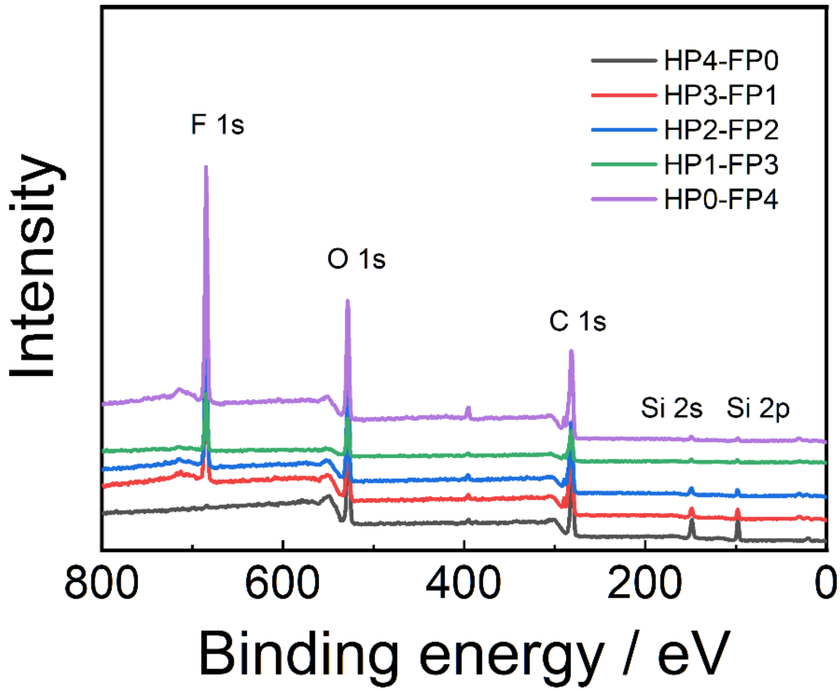

Figure S6. XPS spectra of the HPx-FPy coatings.

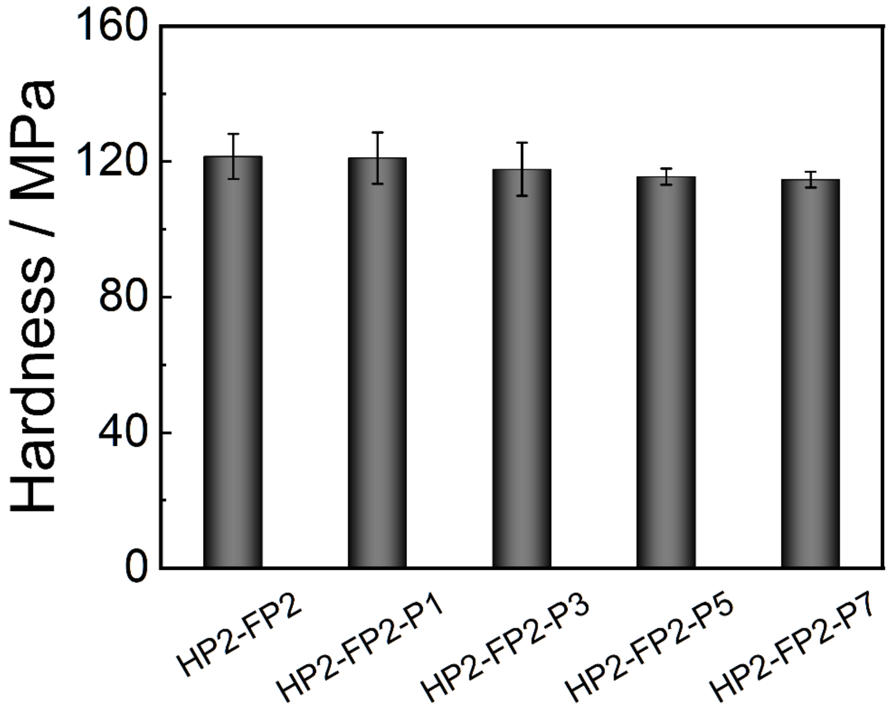

Figure S7. Hardness of the hybrid coatings.

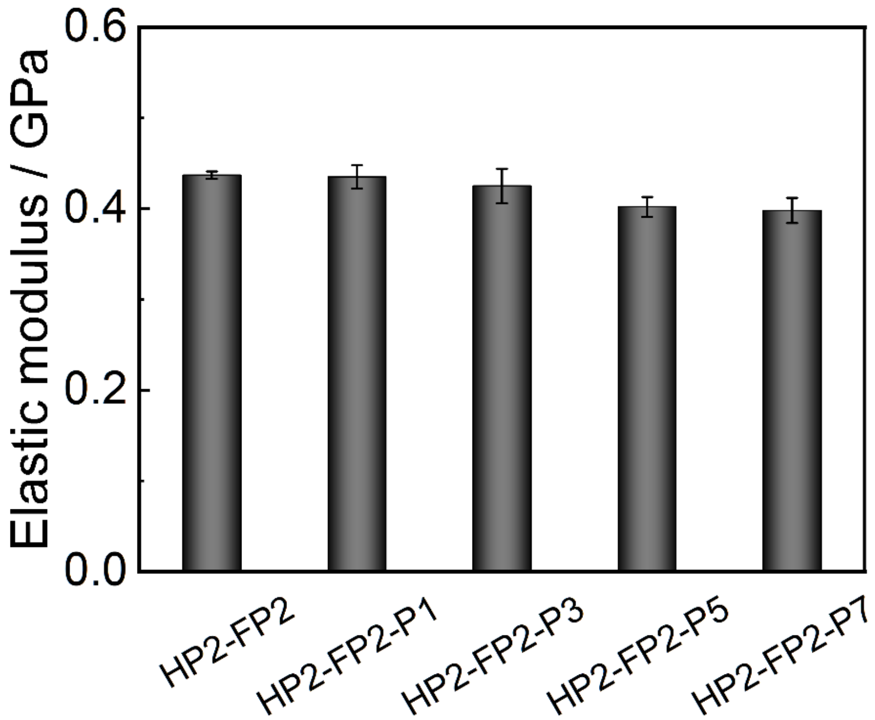

Figure S8. Elastic moduli of the hybrid coatings.

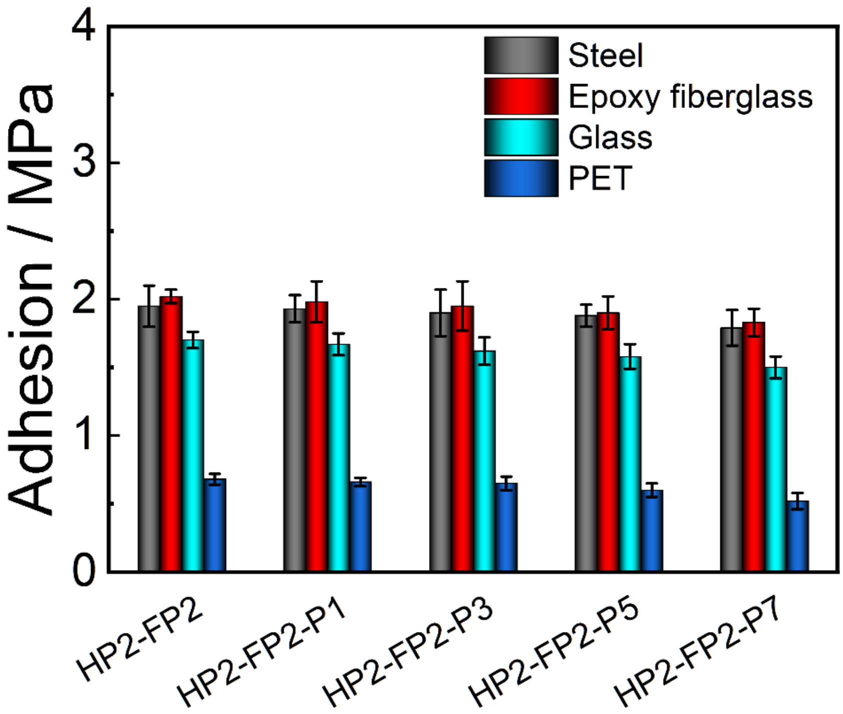

Figure S9. Adhesion strength of the hybrid coatings.

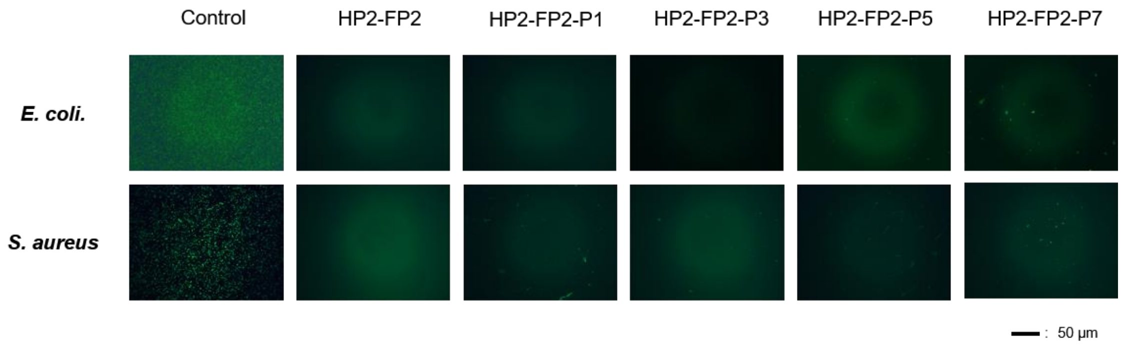

Figure S10. Fluorescence microscopy images of *S. aures* and *E. coli.* on the surface of the hybrid coatings.

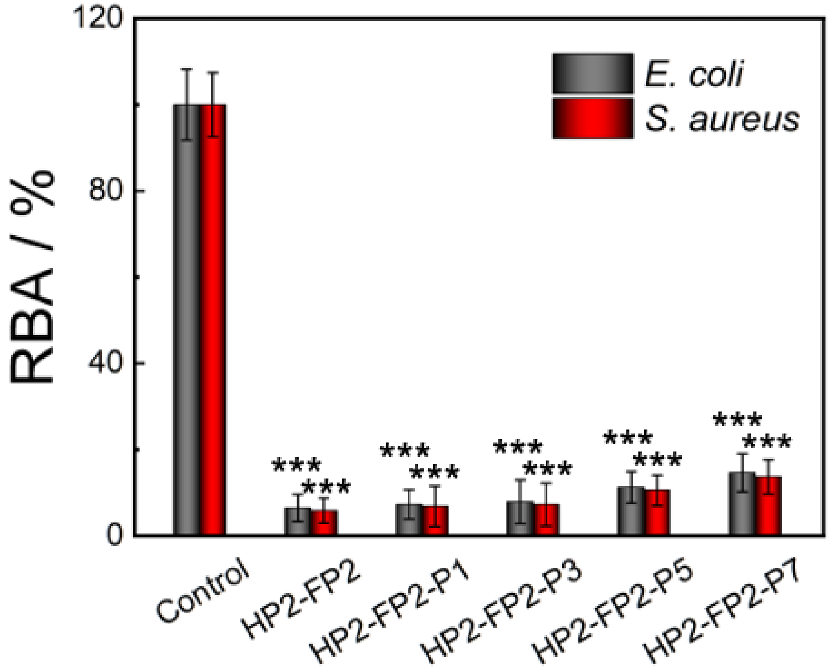

Figure S11. The relative bacteria adhesion (RBA) of the hybrid coatings. ***p < 0.001, compared to the result of silicon wafer (control).

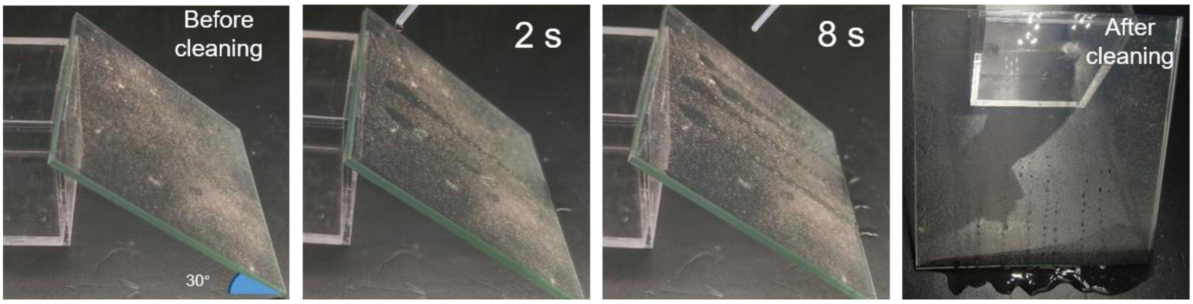

Figure S12. Large-area self-cleaning test of HP2-FP2-P3 coated on a 15×15 cm glass.

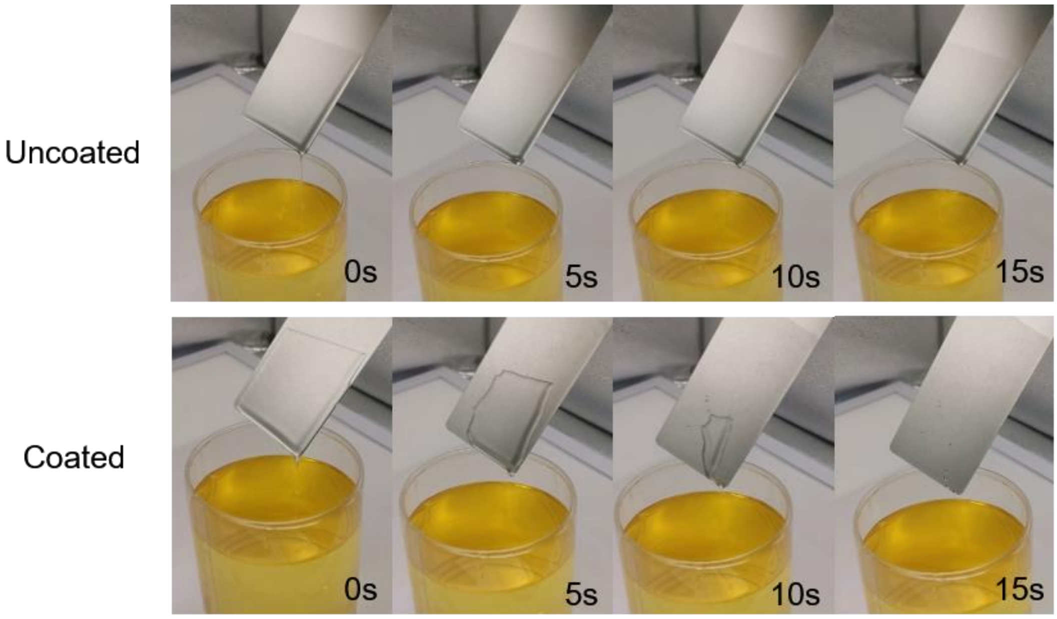

Figure S13. Self-cleaning tests of soybean oil on the uncoated and coated tinplate.

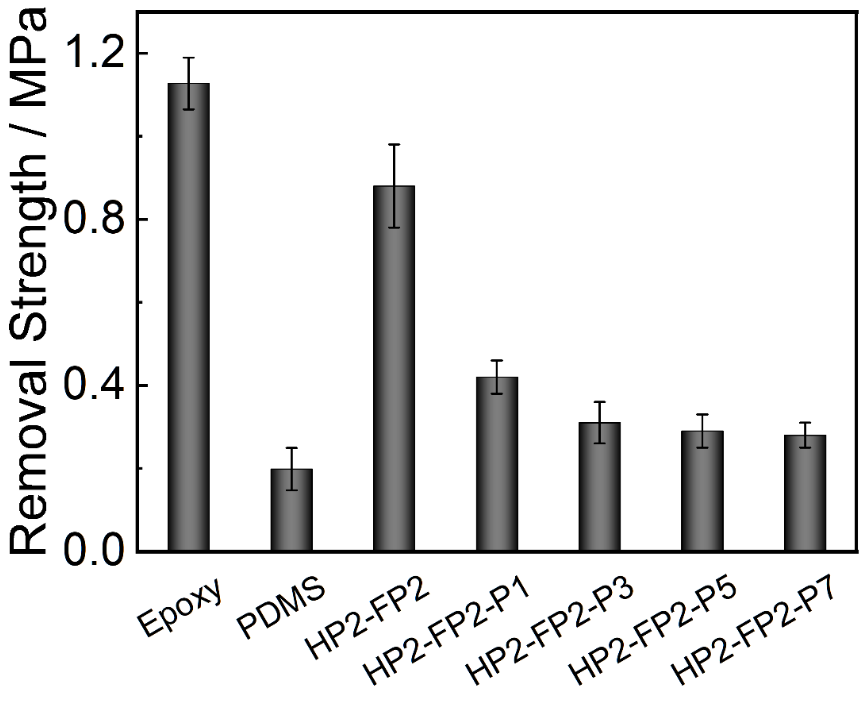

Figure S14. Pseudobarnacle removal strength of epoxy, PDMS, and the hybrid coatings.

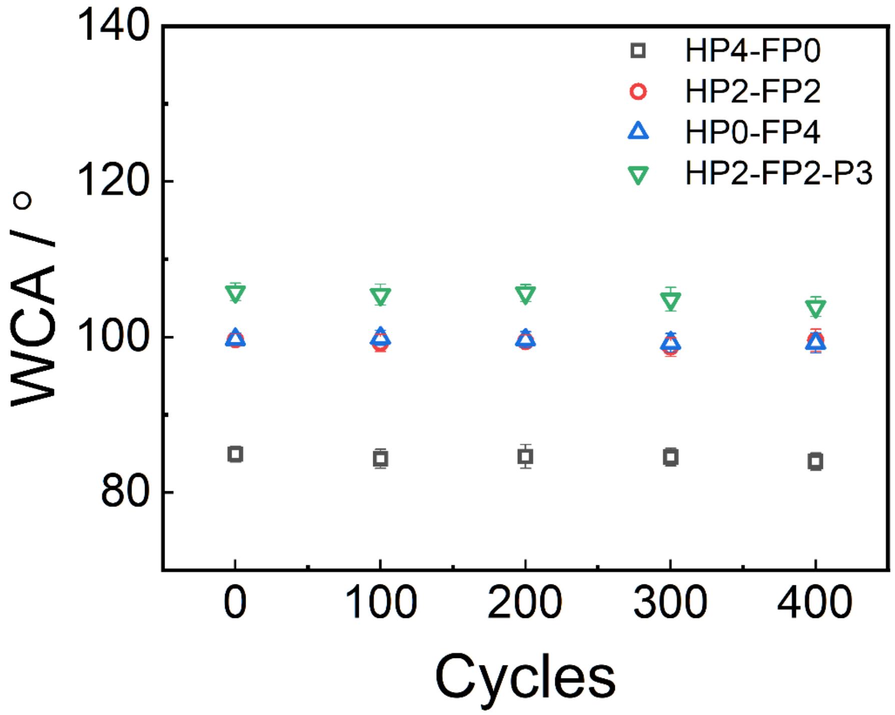

Figure S15. WCA changes of the hybrid coatings after abrasion for 100, 200, 300, and 400 cycles.

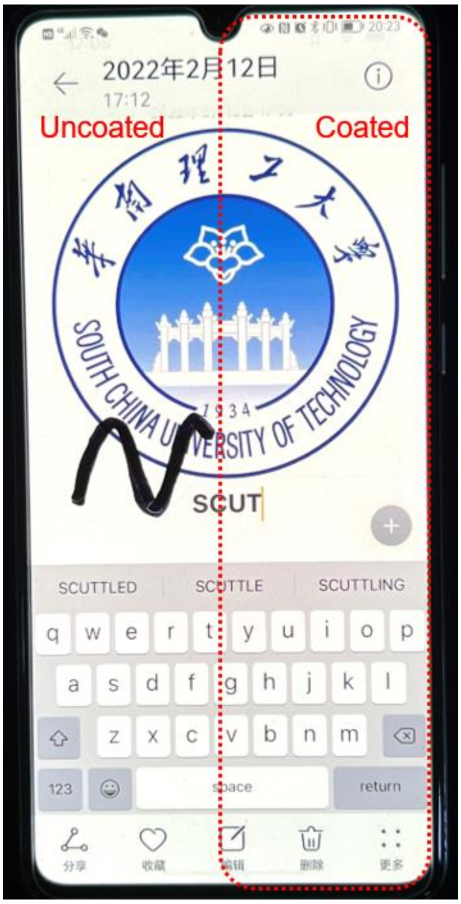

Figure S16. Photograph of HP2-FP2-P3 applied to a mobile phone screen after cleaning.

Table S1. Characterization of the HP-X coatings.

Sample	Curing agent	Drying time	Thickness	Hardness	Bending diameter
HP-D230	D230	2 h	92 ± 6 μm	7 H	15 mm
HP-D400	D400	2 h	94 ± 5 μm	7 H	12 mm
HP-D2000	D2000	12 h	88 ± 3 μm	6 B	3 mm
TPH-D400	D400	3 h	95 ± 5 μm	8 H	12 mm
HP-DDM	DDM	2 h	86 ± 5 μm	8 H	15 mm
HP-MDA	MPD	4 h	96 ± 3 μm	7 H	15 mm
HP-IPDA	IPDA	4 h	98 ± 4 μm	6 H	16 mm

Table S2. The elemental mass content of F on the HPx-FPy coatings.

Sample	F content in bulk	F content on the surface
HP4-FP0	0	0 / 100
HP3-FP1	0.79 / 100	28.84 / 100
HP2-FP2	1.57 / 100	30.91 / 100
HP1-FP3	2.31 / 100	31.59 / 100
HP0-FP4	3.00 / 100	32.94 / 100

Table S3. Characterization of the HPx-FPy coatings.

Sample	Curing agent	Drying time	Thickness	Hardness	Bending diameter
HP4-FP0	D400	2 h	95 ± 5 μm	7 H	12 mm
HP3-FP1	D400	2 h	96 ± 5 μm	7 H	12 mm
HP2-FP2	D400	4 h	94 ± 4 μm	6 H	10 mm
HP1-FP3	D400	4 h	95 ± 3 μm	3 H	10 mm
HP0-FP4	D400	6 h	90 ± 4 μm	H	8 mm

Table S4. Characterization of the hybrid coatings after chemical resistance tests.

Media	HP2-FP2		HP2-FP2-P3	
	Hardness	WCA	Hardness	WCA
UV	6 H	99.0°	6 H	106.2°
Water	6 H	98.4°	6 H	105.4°
Hexane	6 H	98.9°	6 H	105.2°
Xylene	6 H	98.4°	6 H	104.9°
Methanol	6 H	99.4°	6 H	105.8°
Ethanol	6 H	99.3°	6 H	105.9°
0.1 M HCl	6 H	98.4°	6 H	104.5°
0.1 M NaOH	6 H	99.2°	6 H	104.8°

Table S5. Characterization of the HP2-FP2-Pz coatings.

Sample	APT-PDMS/D400[a]	Drying time	Thickness	Hardness	Bending diameter
HP2-FP2-P1	1/99	2 h	93 ± 5 μm	6 H	10 mm
HP2-FP2-P3	3/97	2 h	95 ± 4 μm	6 H	10 mm
HP2-FP2-P5	5/95	2 h	96 ± 5 μm	5 H	10 mm
HP2-FP2-P7	7/93	2 h	90 ± 6 μm	5 H	10 mm

[a]Feed molar ratio.

References

[1] R. Chen, Q. Xie, H. Zeng, C. Ma, G. Zhang. J. Mater. Chem. A 2020, 8, 380.

[2] ASTM D3363-20, Standard Test Method for Film Hardness by Pencil Test, ASTM International, West Conshohocken, PA, 2020.

[3] GB/T 1731-93, Determination of Flexibility of Films, Chinese Standard Publisher: Beijing, China, 1997.

[4] ASTM D4541-09. Standard Test Method for Pull-off Strength of Coatings Using Portable Adhesion Testers; ASTM International: West Conshohocken, PA, 2009.

[5] ASTM G154, Standard Practice for Operating Fluorescent Ultroviolet (UV) Lamp Apparatus for Exposure of Nonmetallic Materials, ASTM International, West Conshohocken, PA, 2016.

[6] ISO 2812-1-2017, Paints and varnishes — Determination of resistance to liquids — Part 1: Immersion in liquids other than water.

[7] Z. Wang, A. A. Volinsky, N. D. Gallant, J. Appl. Polym. Sci. 2015, 132.

[8] ASTM D5618-94, Test Method for Measurement of Barnacle Adhesion Strength in Shear; ASTM International: West Conshohocken, PA, 2011.
